# Decreasing medical complications for total knee arthroplasty: Effect of Critical Pathways on Outcomes

**DOI:** 10.1186/1471-2474-11-160

**Published:** 2010-07-14

**Authors:** M Elaine Husni, Elena Losina, Anne H Fossel, Daniel H Solomon, Nizar N Mahomed, Jeffrey N Katz

**Affiliations:** 1 Department of Rheumatic and Immunologic Diseases, Cleveland Clinic, 9500 Euclid Avenue, Cleveland, OH 44195 USA; 2 Department of Epidemiology and Biostatistics, Boston University School of Public Health, Boston MA 02118 USA; 3 Division of Rheumatology, Immunology and Allergy, Department of Medicine, Brigham and Women's Hospital, 75 Francis Street, Boston, MA 02115, USA; 4 Department of Orthopedic Surgery, Toronto Western Hospital, University Health Network, University of Toronto, 399 Bathurst Street Toronto, ON M5T 2S8, Canada; 5 Department of Orthopedic Surgery, Brigham and Women's Hospital 75 Francis Street, Boston MA 02115 USA

## Abstract

****Background**:**

Studies on critical pathway use have demonstrated decreased length of stay and cost without compromise in quality of care. However, pathway effectiveness is difficult to determine given methodological flaws, such as small or single center cohorts. We studied the effect of critical pathways on total knee replacement outcomes in a large population-based study.

**Methods:**

We identified hospitals in four US states that performed total knee replacements. We sent a questionnaire to surgical administrators in these hospitals including items about critical pathway use and hospital characteristics potentially related to outcomes. Patient data were obtained from Medicare claims, including demographics, comorbidities, 90-day postoperative complications and length of hospital stay. The principal outcome measure was the risk of having one or more postoperative complications.

**Results:**

Two hundred ninety five hospitals (73%) responded to the questionnaire, with 201 reporting the use of critical pathways. 9,157 Medicare beneficiaries underwent TKR in these hospitals with a mean age of 74 years (± 5.8). After adjusting for both patient and hospital related variables, patients in hospitals with pathways were 32% less likely to have a postoperative complication compared to patients in hospitals without pathways (OR 0.68, 95% CI 0.50-0.92). Patients managed on a critical pathway had an average length of stay 0.5 days (95% CI 0.3-0.6) shorter than patients not managed on a pathway.

**Conclusion:**

Medicare patients undergoing total knee replacement surgery in hospitals that used critical pathways had fewer postoperative complications than patients in hospitals without pathways, even after adjusting for patient and hospital related factors.

This study has helped to establish that critical pathway use is associated with lower rates of postoperative mortality and complications following total knee replacement after adjusting for measured variables.

## Background

In the past decade, health care has continued to be a highly regulated environment with fixed reimbursement. The demand is increasing for greater quality and efficiency of heath care. For this reason, critical pathways have gained tremendous popularity for hospital care especially in the surgical arena. Critical pathways (also known by other names such as clinical pathways, care maps, and care paths) are plans of patient care that delineate the sequence of actions necessary to achieve optimal efficiency [1,2]. Critical pathways take many formats, but often are incorporated into daily hospital progress notes either as multi-page forms with space for documentation or as single pages used as checklists of daily items. Despite widespread acceptance of critical pathways for surgical procedures, there has been relatively little rigorous evaluation of the effectiveness of these critical pathways on outcomes [3-6].

Total knee replacement (TKR) presents an ideal model to study the effect of critical pathways on patient outcomes for several reasons. In general, joint replacement is elective and it utilizes a standard treatment protocol with well defined, measurable short-term outcomes. Second, joint replacement patients are considered to be in fairly good health. Third, most joint replacement patients are motivated to return to their usual daily activities. Total knee replacement is one of the most common orthopedic procedures performed and by 2030, the demand for primary total knee arthroplasty may reach greater than 3 million procedures [7].

Early studies on critical pathways and TKR demonstrate a reduction in length of stay (LOS) of up to 57%, and a trend towards decreased cost (savings up to $4,091 per case) without compromise in quality of care [6,8-15] More recent studies by Dy et al report that only seven of 26 pathways used at large academic medical centers have had the desired impact of lowering length of stay [3]. This raises the issue of how critical pathways perform not only within a single hospital but rather in the "bigger picture" of healthcare across the nation. Since the majority of critical pathway studies have focused on length of stay and cost, the effect of critical pathways on patient outcomes is unclear.

Consequently, there is a need for robust research with larger patient samples to effectively examine critical endpoints such as mortality and 90 day postoperative complications. Early studies have not been powered to examine these clinically important outcomes. Previous studies have had numerous methodological limitations including lack of controls or use of historical controls, single hospitals with small samples, limited endpoints such as length of stay rather than patient outcomes, and failure to adjust for potential confounders that may affect outcome [8-13,16,17]. In order to address these gaps in the literature, the influence of critical pathways on the postoperative outcomes of TKR across several hundred hospitals in four states were examined. The analyses adjusted for variables that may confound the association between pathways and patient outcomes including hospital procedure volume.

## Methods

The data used in the paper were obtained from several sources. Medicare claims data were obtained with a Data Use Agreement with the Center for Medicare and Medicaid Services (CMS). The hospital survey data were obtained from by written surveys administered by our research team to administrators at each participating hospital. All of the data collection procedures were approved by the Brigham and Women's/Partners HealthCare Human Investigation Committee.

### Hospital Sample

Using Medicare claims data and previously published algorithms, hospitals in four states were identified (Ohio, Tennessee, North Carolina, and Illinois) that performed at least one total knee replacement in the year 2000 [18]. These states were chosen to encompass diverse geographic locations and a mix of urban and rural areas. Data were collected on the use and implementation of critical pathways in each hospital using a detailed survey that was sent to all the surgical administrators.

The hospital survey contained seven questions about characteristics of critical pathways used for TKR. (See Below) Using information from a literature review and expert consensus, a list of pathway characteristics were identified that might affect pathway effectiveness. These questions permitted classification of the hospitals into three separate categories: no critical pathway used, partial critical pathway used, and full critical pathway used.

List of critical pathway variables that were assessed on the hospital questionnaire

• Uniformity of pathway use among surgeons: Did pathways differ by surgeon?

• Development of clinical pathway: Institution specific or developed by an outside organization

• Critical pathway implementation:

• No pathway used

• Partial pathway in which the hospital used a pathway but did not incorporate it in the medical records

• Full pathway in which the hospital used it for documentation in to the medical records

• Patient access to their pathway: Were they given a copy of the pathway?

• Healthcare providers with specified tasks on the pathway:

• Nursing

• Physical Therapy

• Social Work/care coordination

• Orthopedic surgeon

• Anesthesiologist

• Dietician

### Patient Sample

The patient sample was comprised of Medicare beneficiaries that had an elective total knee replacement in 2000 in hospitals that responded to our survey. Elective cases of primary TKR were identified from Medicare Part A (hospital) and Part B (outpatient and provider) claims using specific surgical procedure and diagnostic codes. The details of patient selection algorithm are described elsewhere [18].

### Hospital Level Data

In addition to using the hospital survey, data were used from the 2000 AHA (American Hospital Association) Annual Survey included the following: whether the hospital was privately or publicly owned; located in a rural or urban setting; a member of the Council of Teaching Hospitals ('teaching hospital') and the Joint Commission on the Accreditation of Health Care Organizations (JCAHO) status. Also data were identified for specific hospital variables that could be associated with clinical outcomes; these included hospital volume, surgeon volume, nurse to patient ratio, and the teaching status of the institution.

Medicare claims provided information on the procedure volume. Hospital and surgeon volume as the total number of primary and revision TKR performed by a surgeon or a hospital in one year was defined. Hospital volume was categorized into tertiles according to the number of TKR procedures performed each year: less than 50, 51-249, and greater than 250 procedures per year. Surgeon volume was categorized into quartiles according to the number of surgeons that operated for total joint arthroplasty in that particular hospital.

### Patient Level Data

Medicare claims data were used to ascertain patient outcomes. These were defined as the occurrence of an adverse event in the first 90 postoperative days including death, myocardial infarction, pneumonia, pulmonary embolus, and deep wound infection (requiring either surgical debridement or removal of the prosthesis). Clinical outcomes were based on diagnosis and procedure codes of inpatient and physician Medicare claims. Medicare claims provided information on age, sex, and eligibility for Medicaid (a surrogate for low income). Comorbid illnesses were defined with an adaptation of the Charlson comorbidity score [19-21].

Length of stay (LOS) was defined as the number of days a patient stayed in the acute care hospital from the date of TKR surgery to the date the patient was discharged home or to a rehabilitation facility.

### **Statistical Methods**

First, bivariate analyses were performed to evaluate crude associations between the use of critical pathways and each postoperative outcome. The principal outcome was a composite dichotomous adverse event indicator that was scored as positive if the patient had any one of the following complications within 90 days of surgery: mortality, myocardial infarction, pneumonia, pulmonary embolus, and deep wound infection. A multivariable logistic regression model was constructed to evaluate the odds of a postoperative complication or death in the pathway and non pathway groups, adjusting for patient age, gender, co-morbidity index, Medicaid eligibility (a proxy for poverty level income) and hospital volume. Specific hospital variables that could potentially be associated with clinical outcomes were assessed; these included surgeon volume, JCAHO accreditation, and presence of an orthopedic residency program, nurse to patient ratio, and the teaching status of the institution. The association between use of critical pathway and outcome was expressed with an adjusted odds ratio. Generalized estimating equations were used to adjust for clustering within hospitals for all models [22]. A sensitivity analysis was performed to further delineate whether level of critical pathway implementation affected outcome. The lengths of stay between those patients with and without a critical pathway were also examined.

## Results

### Hospital Sample

**Figure 1:**
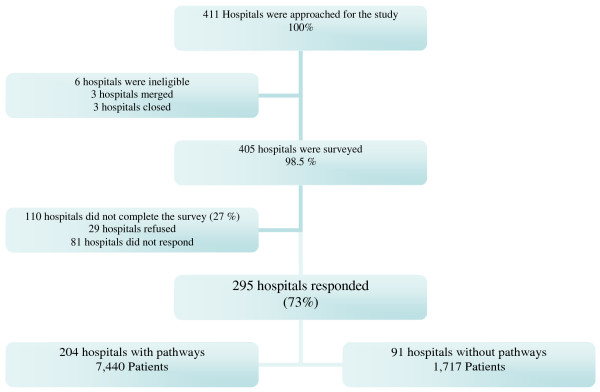
**Schema of hospitals and patients that participated in the study**.

411 hospitals in Ohio, Tennessee, North Carolina, and Illinois were approached. Six hospitals were excluded due to mergers (3 hospitals) and closings (3 hospitals) in 2000 leaving 405 eligible hospitals. Of these, 295 (73%) hospitals completed the survey, 29 (7%) hospitals refused to participate while an additional 81 (20%) hospitals did not respond to follow up letters or calls. (See Figure 1) There was no statistically significant difference in hospital characteristics between hospitals that responded to the survey and those that did not in terms of rural or urban setting, teaching status, or private or public ownership. Of the 295 hospitals surveyed, 201 (68%) reported using a critical pathway for TKR surgery.

Patient Cohort: A total of 9,291 Medicare patients had primary TKR at the hospitals that completed the survey. 134 patients were excluded due to missing information on patient variables leaving 9,157 patients in the final cohort. Patients on pathways differed slightly from patients not on pathways in terms of Medicaid eligibility, a proxy for low income. In the non pathway group, 8.6% were eligible for Medicaid vs. 6.4% in the pathway group (p value = 0.037). In both the critical pathway and non critical pathway cohorts, mean age was 74 years, 31% were male, and > 30% had one or more co morbidities. (See Table 1)

**Table 1:** Characteristics of Patients and Hospitals With and Without A Critical Pathway

Characteristics	Critical Pathway N (%)	No Critical Pathway N (%)	P value
**Patients**	7,440 (81.2)	1,717 (18.8)	
Age, mean (SD), years	74.3 (+/-5.8)	73.9(+/-5.7)	NS
Gender, male	2,276(31)	535(31)	NS
Number of comorbid illnesses			
0	4680 (63)	1,116(65)	NS
1	1741 (23)	378(22)	NS
> 1	1019(14)	223(13)	NS
Medicaid eligible	476(6.4)	149(8.6)	p = 0.04
Adverse events			
Death	40(0.5)	18 (1.1)	p = 0.02
Acute myocardial infarction	59(0.8)	22 (1.3)	p = 0.05
Pneumonia	102 (1.4)	37 (2.2)	p = 0.02
Pulmonary embolus	58(0.8)	22 (1.3)	p = 0.04
Deep wound infection	26 (.4)	6 (.4)	NS
**Hospitals**	**Critical** **Pathway ** N = 204	**No Critical** **Pathway** N = 91	
Length of Stay	5.8 (2.4)	5.3 (2.0)	p = .00001

### Description of pathway use

**Figure 2:**
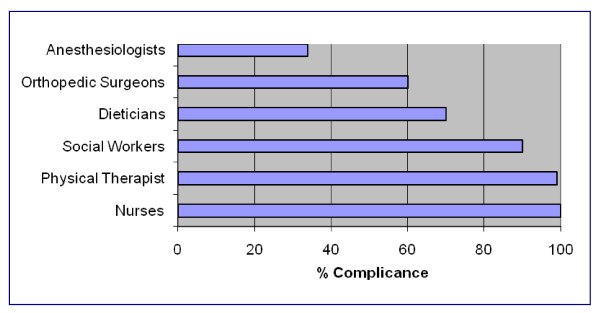
**Percentage Compliance and Categories of Care in Hospitals with Critical Pathways**.

The majority of hospitals that used pathways developed their own pathway (87%) while others either modified a pathway from an outside source (7.8%) or used a pathway from an outside source without (1.4%) modification. We also queried whether patients were all managed on the same pathway or different pathways for each surgeon; 60% of hospitals stated patients were managed on the same pathway and 11% stated patients were managed by different pathways according to the surgeon. About half or 55% of the hospitals stated they gave patients a copy of the critical pathway during their hospitalization. Almost all critical pathways had duties specific to nurses (100%), physical therapists (99%), social workers (90%), and dieticians (70%). (See Figure 2) Interestingly, only a small percentage of pathways had specific duties for physicians. For example, only 60% of orthopedic surgeons and 34% of anesthesiologists had specified duties on the critical pathways postoperatively as compared with 100% for nurses and 99% for physical therapists.

### Primary analyses of the effect of pathway use on outcomes

The primary analyses demonstrated that patients operated upon in pathway hospitals had a lower risk of postoperative complications including death compared to patients operated upon in hospitals without pathways. Table 2 illustrates the results of logistic regression analyses, which controlled for socio demographic factors, hospital and surgeon volume, and hospital teaching status. The regression results showed a statistically significant relationship between critical pathway use and a lower risk of specific postoperative patient outcomes. With the exception of infection, the odds ratios were less than one ranging from 0.55 (95% CI 0.32-0.93) for mortality to 0.66 (95% CI 0.43-1.02) for pneumonia suggesting a protective effect of pathways on patient outcome. Since the prevalence of postoperative complications after TKR surgery is low, a composite adverse event indicator was developed which scored positive if the patient had *any *one of the adverse events. These analyses showed that there were 32% fewer adverse events in patients on critical pathways as compared to those without a critical pathway (adjusted OR = 0.68, 95% CI 0.50, 0.92).

**Table 2:** Crude and Adjusted odds ratios for effect of pathway on select patient outcomes *

Outcome	% Observed events with pathway	% Observed events without pathway	Crude OR (95%CI)	Adjusted OR (95%CI)
Mortality	0.54	1.05	0.51 (0.29-0.89)	**0.55 (0.32-0.93)**
Myocardial Infarction	0.79	1.28	0.62 (0.38-1.0)	0.63 (0.37-1.08)
Pneumonia	1.37	2.15	0.63 (0.43-0.92)	0.66(0.43-1.02)
Pulmonary Embolus	0.78	1.28	0.61 (0.37-0.99)	0.59 (0.35-1.01)
Infection	0.35	0.35	1.0 (0.41-2.4)	0.98 (0.43-2.24)
Composite	3.43	4.83	0.69 (0.54-0.90)	**0.68 (0.50-0.92)**

*Adjusted for both patient level (age, gender, Medicaid eligibility) and hospital level (hospital volume, surgeon volume, hospital characteristics) factors. Composite score was defined as having any one of the mentioned perioperative complications listed.

Adjusted length of stay was an average of 0.5 days (95% CI 0.3-0.6) shorter for patients on critical pathways compared with patients without a critical pathway. These analyses were adjusted for both patient level (age, gender, Medicaid eligibility) and hospital level (hospital volume, surgeon volume, hospital characteristics) factors.

### Additional analyses of pathway implementation strategies

Of the 201 hospitals using a critical pathway, the level of implementation varied with 38% (76/201) of hospitals using the partial critical pathway category (pathways used but not incorporated into medical records), and 64% (128/201) of hospitals use the full critical pathway category (pathways used and incorporated into medical records). More detailed analyses were performed to examine the effect of the level of critical pathway implementation. These analyses did not demonstrate an association between level of pathway implementation and patient outcomes with exception of pneumonia (p-value for trend 0.006).

## Discussion

The association between critical pathway use and 90 day adverse event rates in a population based sample of patients undergoing TKR was examined. The findings indicate that postoperative complication rates were lower in patients operated upon in hospitals that used critical pathways compared to patients in hospitals without critical pathways, suggesting a beneficial effect of critical pathways on patient outcomes. Length of stay was also shortened for those patients in hospitals that used critical pathways by 0.5 days. To our knowledge, this work on the effect of critical pathway use on TKR outcomes represents the largest, most geographically diverse, and comprehensive study of the outcomes of critical pathways to date. Prior studies of critical pathways were limited by small samples, restriction to one or a few referral centers, lack of controls, or the use of historical controls limiting the ability to generalize and/or introduced bias [4-6,24-26].

The finding of improved patient outcomes associated with pathway use raises questions about the mechanisms whereby critical pathways may affect outcomes. Do pathways increase the use of proven medical therapies for patients? Do pathways ensure that organization and delivery of care to each TKR patient is consistent? Do pathways allow multidisciplinary teams to discuss each patient with accountability? These questions cannot satisfactorily be answered with our data but, raise these issues to advance the understanding of critical pathways. Key hospital characteristics that may have affected outcomes, such as physical therapy availability and standardization, specific discharge status, and orthopedic operating room standards were not measured at the patient level. Adjustments for patient demographics and clinical characteristics were examined; these factors did not change the finding that hospitals using pathways had lower rates of complications. Given the positive association of outcomes on patients following critical pathways, future studies may benefit from emphasis on specific processes of care that effect outcome. Future work on critical pathways should expand outcomes to include quality of life, satisfaction, and adherence to pathway management.

This study echoed results of previously published papers reporting a shorter length of stay with patients on a critical pathway compared to those not on a critical pathway as performed by an English language MEDLINE search in April 2007 [1-8,12-14,26,27]. Even with adjustment for patient demographics and hospital volume, patients on a critical pathway had an average of 0.5 days shorter length of stay compared to those not on a pathway. However, this difference of 0.5 days may not be meaningful if in fact it was achieved at the expense of increased rehabilitation stay for these patients at another facility [4-26]. Further research should be done to clarify whether pathways truly decrease costs or simply shift them.

A pilot study was performed to better understand pathways used by different institutions. Pathways from 40 hospitals were obtained in the sample and noted tremendous variability in content and level of detail. For example, pathway length ranged from 2 to 18 pages in this sample of 40. However, the majority of critical pathways examined shared key elements including specific process of care (specialized pre op teaching session, pre specified schedule of patient care including physical therapy, deep vein thrombosis prophylaxis, Foley urinary catheter removal, and nursing care) and a multidisciplinary approach including the use of specialty services such as physical therapy, dieticians, and social workers and discharge planning checklist. Most variations on critical pathways were attributed to level of detail and number of days to be followed. Some critical pathways accounted for every action that could possibly occur in the care process so that nurses can merely check off boxes after each item was accomplished. Other critical pathways accounted for pre operative day 1 or 2 leaving the rest of the hospitalization to traditional, daily progress notes.

The main strengths of this study include its large population based sample and ability to capture outcomes assessed with standardized, previously validated algorithms. Further, 90 day adverse events were irrespective of whether they occurred in the acute hospitals, at a rehabilitation facility or at home. The analyses adjusted for potential confounders including patient characteristics and were simultaneously adjusted for both hospital and surgeon volume. Adjustment was also made for clustering of patients within hospitals.

An important limitation is that this study was cross sectional. Thus, causality, such as whether pathways lead to better outcomes or vice versa cannot be determined. The use of the Medicare database may also present potential limitations. These include confining the study to patients greater than 65 years of age. However, more than two thirds of TKR procedures are generally performed in people over the age of 65 [7]. In addition, Medicare claims do not provide data on functional status of the patient, an important outcome of TKR. Finally, it would be useful to examine the effects of clinical pathways implemented in the rehabilitation process following TKR. This was beyond the scope of our data and remains an important research priority.

In conclusion, this study has established that critical pathway use is associated with lower rates of postoperative mortality and complications following total knee replacement after adjusting for measured variables. Further study is required to address mechanistic questions raised by the current findings; to identify the patient- provider, and system-level factors that drive this effect. In the meantime, these data should be taken into account by hospitals considering whether to implement critical pathways for TKR.

## Competing interests

The authors declare that they have no competing interests.

## Authors' contributions

MEH, JK conceived of the study, participated in the design and coordination of the study, assisted in the design of the statistical analysis, and interpretation of the data. MEH wrote the first draft of the manuscript. EL, JK participated in the design and performed the statistical analysis, analyzed and interpreted the data, and provided the critical review of the manuscript for important intellectual content. AF, MEH was responsible for the acquisition of data, coordination of the study, and manuscript preparation. DS, NM participated in the design of the study, interpretation of the data, and manuscript preparation. All authors were involved in preparation and revising the manuscript and have read and approved the final manuscript

## Pre-publication history

The pre-publication history for this paper can be accessed here:

http://www.biomedcentral.com/1471-2474/11/160/prepub
